# Supplementary material for: Origins and geographic diversification of African rice (Oryza glaberrima)
Source: PLoS One. 2019 Mar 6;14(3):e0203508. doi: 10.1371/journal.pone.0203508 (PMC6402627; doi:10.1371/journal.pone.0203508)
Supplement: S1 Table — (PDF) [file pone.0203508.s001.pdf]

S1 Table. List of accessions included in this study. *Oryza glaberrima* data first published in Wang et al. [1], Meyer et al. [2] and Van Andel et al. [3].

Species	Accession number	Collection	Country	Latitude	Longitude	Source publication	SRA study	SRA run
Oryza glaberrima	IRGC101049	International Rice Research Institute	South Africa	-33.6106	26.83333	Wang et al. (2014)	SRP038750	SRR1206507
Oryza glaberrima	IRGC103442	International Rice Research Institute	Senegal	12.65	-15.4667	Meyer et al. (2016)	SRP071857	SRR3231659
Oryza glaberrima	IRGC103450	International Rice Research Institute	Gambia	13.25	-15.8333	Meyer et al. (2016)	SRP071857	SRR3231660
Oryza glaberrima	IRGC103452	International Rice Research Institute	Senegal	13.76639	-16.4833	Meyer et al. (2016)	SRP071857	SRR3231661
Oryza glaberrima	IRGC103456	International Rice Research Institute	Senegal	13.01667	-15.7167	Meyer et al. (2016)	SRP071857	SRR3231662
Oryza glaberrima	IRGC103461	International Rice Research Institute	Senegal	13.53333	-16.4167	Meyer et al. (2016)	SRP071857	SRR3231663
Oryza glaberrima	IRGC103463	International Rice Research Institute	Senegal	12.51667	-16.5833	Meyer et al. (2016)	SRP071857	SRR3231664
Oryza glaberrima	IRGC103469	International Rice Research Institute	Burkina Faso	11.33333	-4.41667	Wang et al. (2014)	SRP038750	SRR1206500
Oryza glaberrima	IRGC103472	International Rice Research Institute	Burkina Faso	10.53333	-4.76667	Wang et al. (2014)	SRP038750	SRR1206508
Oryza glaberrima	IRGC103517	International Rice Research Institute	Mali	14.33333	-4.9	Meyer et al. (2016)	SRP071857	SRR3231665
Oryza glaberrima	IRGC103520	International Rice Research Institute	Mali	14.33333	-4.9	Wang et al. (2014)	SRP038750	SRR1206509
Oryza glaberrima	IRGC103530	International Rice Research Institute	Mali	14.23333	-3.61667	Meyer et al. (2016)	SRP071857	SRR3231666
Oryza glaberrima	IRGC103592	International Rice Research Institute	Cameroon	10.16667	14.33333	Meyer et al. (2016)	SRP071857	SRR3231667
Oryza glaberrima	IRGC103599	International Rice Research Institute	Cameroon	8.7	14.18333	Meyer et al. (2016)	SRP071857	SRR3231668
Oryza glaberrima	IRGC103632	International Rice Research Institute	Mali	14.88333	-3.99139	Wang et al. (2014)	SRP038750	SRR1206510
Oryza glaberrima	IRGC103922	International Rice Research Institute	Nigeria	11.16667	4.66667	Meyer et al. (2016)	SRP071857	SRR3231669
Oryza glaberrima	IRGC103937	International Rice Research Institute	Liberia	6.18333	-9.76667	Meyer et al. (2016)	SRP071857	SRR3231670
Oryza glaberrima	IRGC103946	International Rice Research Institute	Liberia	8.4	-10.1833	Meyer et al. (2016)	SRP071857	SRR3231671
Oryza glaberrima	IRGC103948	International Rice Research Institute	Liberia	8.18333	-10.2333	Meyer et al. (2016)	SRP071857	SRR3231672
Oryza glaberrima	IRGC103949	International Rice Research Institute	Liberia	8.28333	-10.1	Meyer et al. (2016)	SRP071857	SRR3231673
Oryza glaberrima	IRGC103953	International Rice Research Institute	Sierra Leone	9	-13	Meyer et al. (2016)	SRP071857	SRR3231674
Oryza glaberrima	IRGC103955	International Rice Research Institute	Senegal	12.62972	-16.0167	Meyer et al. (2016)	SRP071857	SRR3231675
Oryza glaberrima	IRGC103956	International Rice Research Institute	Senegal	12.58583	-15.5433	Meyer et al. (2016)	SRP071857	SRR3231676
Oryza glaberrima	IRGC103957	International Rice Research Institute	Senegal	12.60083	-16.0694	Meyer et al. (2016)	SRP071857	SRR3231677
Oryza glaberrima	IRGC103958	International Rice Research Institute	Senegal	12.63639	-15.4725	Meyer et al. (2016)	SRP071857	SRR3231678
Oryza glaberrima	IRGC103959	International Rice Research Institute	Senegal	12.55556	-15.8275	Meyer et al. (2016)	SRP071857	SRR3231679
Oryza glaberrima	IRGC103960	International Rice Research Institute	Senegal	12.57028	-15.9042	Meyer et al. (2016)	SRP071857	SRR3231680

Species	Accession number	Collection	Country	Latitude	Longitude	Source publication	SRA study	SRA run
Oryza glaberrima	IRGC103963	International Rice Research Institute	Senegal	12.52944	-15.7081	Meyer et al. (2016)	SRP071857	SRR3231681
Oryza glaberrima	IRGC103967	International Rice Research Institute	Senegal	12.50083	-15.5217	Meyer et al. (2016)	SRP071857	SRR3231682
Oryza glaberrima	IRGC103981	International Rice Research Institute	Nigeria	8.91667	4.63333	Meyer et al. (2016)	SRP071857	SRR3231683
Oryza glaberrima	IRGC103982	International Rice Research Institute	Nigeria	12.75	8.83333	Meyer et al. (2016)	SRP071857	SRR3231684
Oryza glaberrima	IRGC103988	International Rice Research Institute	Sierra Leone	8.28333	-10.5833	Meyer et al. (2016)	SRP071857	SRR3231685
Oryza glaberrima	IRGC103989	International Rice Research Institute	Sierra Leone	8.23333	-10.3667	Meyer et al. (2016)	SRP071857	SRR3231686
Oryza glaberrima	IRGC103991	International Rice Research Institute	Sierra Leone	8.13333	-10.8833	Meyer et al. (2016)	SRP071857	SRR3231687
Oryza glaberrima	IRGC103992	International Rice Research Institute	Sierra Leone	7.28333	-11.3333	Meyer et al. (2016)	SRP071857	SRR3231688
Oryza glaberrima	IRGC103993	International Rice Research Institute	Sierra Leone	9.75	-12.5	Meyer et al. (2016)	SRP071857	SRR3231689
Oryza glaberrima	IRGC103994	International Rice Research Institute	Sierra Leone	9.05	-11.7333	Meyer et al. (2016)	SRP071857	SRR3231690
Oryza glaberrima	IRGC103995	International Rice Research Institute	Sierra Leone	9.11667	-11.9167	Meyer et al. (2016)	SRP071857	SRR3231691
Oryza glaberrima	IRGC104011	International Rice Research Institute	Nigeria	8.88333	11.38333	Meyer et al. (2016)	SRP071857	SRR3231692
Oryza glaberrima	IRGC104022	International Rice Research Institute	Guinea-Bissau	11.785	-15.9175	Meyer et al. (2016)	SRP071857	SRR3231693
Oryza glaberrima	IRGC104023	International Rice Research Institute	Guinea-Bissau	11.88333	-15.8497	Meyer et al. (2016)	SRP071857	SRR3231694
Oryza glaberrima	IRGC104024	International Rice Research Institute	Guinea-Bissau	11.86917	-15.6086	Meyer et al. (2016)	SRP071857	SRR3231695
Oryza glaberrima	IRGC104025	International Rice Research Institute	Guinea-Bissau	12.07917	-15.3214	Meyer et al. (2016)	SRP071857	SRR3231696
Oryza glaberrima	IRGC104028	International Rice Research Institute	Guinea-Bissau	12.34861	-14.3528	Meyer et al. (2016)	SRP071857	SRR3231697
Oryza glaberrima	IRGC104029	International Rice Research Institute	Guinea-Bissau	12.16667	-14.6664	Meyer et al. (2016)	SRP071857	SRR3231698
Oryza glaberrima	IRGC104030	International Rice Research Institute	Guinea-Bissau	11.68333	-14.7658	Meyer et al. (2016)	SRP071857	SRR3231699
Oryza glaberrima	IRGC104032	International Rice Research Institute	Guinea-Bissau	11.35583	-15.1167	Meyer et al. (2016)	SRP071857	SRR3231700
Oryza glaberrima	IRGC104034	International Rice Research Institute	Cote d'Ivoire	7.316944	-7.5125	Meyer et al. (2016)	SRP071857	SRR3231701
Oryza glaberrima	IRGC104035	International Rice Research Institute	Cote d'Ivoire	6.685556	-8.31139	Meyer et al. (2016)	SRP071857	SRR3231702
Oryza glaberrima	IRGC104036	International Rice Research Institute	Cote d'Ivoire	6.589722	-8.24583	Meyer et al. (2016)	SRP071857	SRR3231703
Oryza glaberrima	IRGC104044	International Rice Research Institute	Chad	9.41667	16.33333	Meyer et al. (2016)	SRP071857	SRR3231704
Oryza glaberrima	IRGC104047	International Rice Research Institute	Cameroon	10.75	13.83333	Meyer et al. (2016)	SRP071857	SRR3231705
Oryza glaberrima	IRGC104165	International Rice Research Institute	Guinea	8.381944	-9.29944	Meyer et al. (2016)	SRP071857	SRR3231706
Oryza glaberrima	IRGC104173	International Rice Research Institute	Guinea	10.01639	-10.8333	Meyer et al. (2016)	SRP071857	SRR3231707
Oryza glaberrima	IRGC104177	International Rice Research Institute	Guinea	11.07139	-14.4219	Meyer et al. (2016)	SRP071857	SRR3231708
Oryza glaberrima	IRGC104178	International Rice Research Institute	Guinea	12.35472	-13.14	Meyer et al. (2016)	SRP071857	SRR3231709

Species	Accession number	Collection	Country	Latitude	Longitude	Source publication	SRA study	SRA run
Oryza glaberrima	IRGC104180	International Rice Research Institute	Guinea	11.18306	-12.2042	Meyer et al. (2016)	SRP071857	SRR3231710
Oryza glaberrima	IRGC104181	International Rice Research Institute	Guinea	10.89861	-12.5586	Meyer et al. (2016)	SRP071857	SRR3231711
Oryza glaberrima	IRGC104182	International Rice Research Institute	Guinea	10.81667	-12.6997	Meyer et al. (2016)	SRP071857	SRR3231712
Oryza glaberrima	IRGC104187	International Rice Research Institute	Guinea	10.91639	-12.1833	Meyer et al. (2016)	SRP071857	SRR3231713
Oryza glaberrima	IRGC104190	International Rice Research Institute	Guinea	10.58417	-12.5608	Meyer et al. (2016)	SRP071857	SRR3231714
Oryza glaberrima	IRGC104194	International Rice Research Institute	Guinea	9.273056	-13.0242	Meyer et al. (2016)	SRP071857	SRR3231715
Oryza glaberrima	IRGC104195	International Rice Research Institute	Ghana	6.15	0.26667	Meyer et al. (2016)	SRP071857	SRR3231716
Oryza glaberrima	IRGC104206	International Rice Research Institute	Ghana	7.63333	0.83333	Wang et al. (2014)	SRP038750	SRR1206512
Oryza glaberrima	IRGC104231	International Rice Research Institute	Sierra Leone	7.83333	-10.7667	Meyer et al. (2016)	SRP071857	SRR3231717
Oryza glaberrima	IRGC104260	International Rice Research Institute	Ghana	7.23333	0.56667	Meyer et al. (2016)	SRP071857	SRR3231718
Oryza glaberrima	IRGC104294	International Rice Research Institute	Chad	9.66667	15	Meyer et al. (2016)	SRP071857	SRR3231719
Oryza glaberrima	IRGC104533	International Rice Research Institute	Nigeria	12.83333	4.7	Meyer et al. (2016)	SRP071857	SRR3231720
Oryza glaberrima	IRGC104545	International Rice Research Institute	Nigeria	15.83333	5.83333	Meyer et al. (2016)	SRP071857	SRR3231721
Oryza glaberrima	IRGC104561	International Rice Research Institute	Sierra Leone	8.26667	-10.4833	Meyer et al. (2016)	SRP071857	SRR3231722
Oryza glaberrima	IRGC104562	International Rice Research Institute	Sierra Leone	9.66667	-11.5833	Meyer et al. (2016)	SRP071857	SRR3231723
Oryza glaberrima	IRGC104566	International Rice Research Institute	Senegal	12.71667	-15.6	Meyer et al. (2016)	SRP071857	SRR3231724
Oryza glaberrima	IRGC104571	International Rice Research Institute	Senegal	14.49944	-14.4456	Meyer et al. (2016)	SRP071857	SRR3231725
Oryza glaberrima	IRGC104573	International Rice Research Institute	Cote d'Ivoire	9.2	-3.03333	Meyer et al. (2016)	SRP071857	SRR3231726
Oryza glaberrima	IRGC104574	International Rice Research Institute	Mali	12.4	-5.4	Wang et al. (2014)	SRP038750	SRR1206513
Oryza glaberrima	IRGC104595	International Rice Research Institute	Mali	12.3	-7.93333	Meyer et al. (2016)	SRP071857	SRR3231727
Oryza glaberrima	IRGC104904	International Rice Research Institute	Nigeria	10.5	4.66667	Meyer et al. (2016)	SRP071857	SRR3231728
Oryza glaberrima	IRGC104934	International Rice Research Institute	Burkina Faso	10.83333	-4.58333	Meyer et al. (2016)	SRP071857	SRR3231729
Oryza glaberrima	IRGC104955	International Rice Research Institute	Sierra Leone	9.5	-12.2333	Wang et al. (2014)	SRP038750	SRR1206514
Oryza glaberrima	IRGC105005	International Rice Research Institute	Guinea	11.67667	-9.42556	Meyer et al. (2016)	SRP071857	SRR3231730
Oryza glaberrima	IRGC105011	International Rice Research Institute	Guinea	11.43333	-9.03306	Meyer et al. (2016)	SRP071857	SRR3231731
Oryza glaberrima	IRGC105021	International Rice Research Institute	Guinea	10.84972	-10.9333	Meyer et al. (2016)	SRP071857	SRR3231732
Oryza glaberrima	IRGC105026	International Rice Research Institute	Guinea	9.430556	-13.0878	Meyer et al. (2016)	SRP071857	SRR3231733
Oryza glaberrima	IRGC105034	International Rice Research Institute	Guinea	10.34972	-14.3664	Meyer et al. (2016)	SRP071857	SRR3231734
Oryza glaberrima	IRGC105036	International Rice Research Institute	Guinea	10.66611	-14.5997	Meyer et al. (2016)	SRP071857	SRR3231735

Species	Accession number	Collection	Country	Latitude	Longitude	Source publication	SRA study	SRA run
Oryza glaberrima	IRGC105038	International Rice Research Institute	Guinea	9.949722	-12.9333	Meyer et al. (2016)	SRP071857	SRR3231736
Oryza glaberrima	IRGC105043	International Rice Research Institute	Guinea	11.21667	-11.8164	Meyer et al. (2016)	SRP071857	SRR3231737
Oryza glaberrima	IRGC105044	International Rice Research Institute	Guinea	11.31667	-12.2831	Meyer et al. (2016)	SRP071857	SRR3231738
Oryza glaberrima	IRGC105048	International Rice Research Institute	Liberia	6.98333	-9.6	Meyer et al. (2016)	SRP071857	SRR3231739
Oryza glaberrima	IRGC105049	International Rice Research Institute	Liberia	6.98333	-9.58333	Meyer et al. (2016)	SRP071857	SRR3231740
Oryza glaberrima	IRGC105050	International Rice Research Institute	Liberia	6.23333	-9.93333	Meyer et al. (2016)	SRP071857	SRR3231741
Oryza glaberrima	IRGC105052	International Rice Research Institute	Guinea	10.18333	-14.0664	Meyer et al. (2016)	SRP071857	SRR3231742
Oryza glaberrima	IRGC58622	International Rice Research Institute	Sierra Leone	8.464444	-11.7958	Meyer et al. (2016)	SRP071857	SRR3231743
Oryza glaberrima	IRGC61457	International Rice Research Institute	Liberia	6.452222	-9.42833	Meyer et al. (2016)	SRP071857	SRR3231744
Oryza glaberrima	IRGC67563	International Rice Research Institute	Ghana	6.719167	0.526111	Meyer et al. (2016)	SRP071857	SRR3231745
Oryza glaberrima	IRGC68939	International Rice Research Institute	Madagascar	-18.7772	46.83111	Wang et al. (2014)	SRP038750	SRR1206516
Oryza glaberrima	IRGC68976	International Rice Research Institute	Guyana	4.866111	-58.9381	Wang et al. (2014)	SRP038750	SRR1206517
Oryza glaberrima	IRGC75500	International Rice Research Institute	Burkina Faso	12.9	-2.44972	Wang et al. (2014)	SRP038750	SRR1206518
Oryza glaberrima	IRGC75546	International Rice Research Institute	Burkina Faso	13.65278	-0.55083	Meyer et al. (2016)	SRP071857	SRR3231746
Oryza glaberrima	IRGC75618	International Rice Research Institute	Burkina Faso	12.01639	-2.31639	Meyer et al. (2016)	SRP071857	SRR3231747
Oryza glaberrima	IRGC75729	International Rice Research Institute	Burkina Faso	12.74528	-3.76306	Meyer et al. (2016)	SRP071857	SRR3231748
Oryza glaberrima	IRGC96841	International Rice Research Institute	Zimbabwe	-19.0131	29.14639	Wang et al. (2014)	SRP038750	SRR1206519
Oryza glaberrima	TOG5457	Africa Rice Center	Nigeria	11.93333	4.18333	Wang et al. (2014)	SRP038750	SRR1206501
Oryza glaberrima	TOG5467	Africa Rice Center	Nigeria	N.A.	N.A.	Wang et al. (2014)	SRP038750	SRR1206502
Oryza glaberrima	TOG5923	Africa Rice Center	Liberia	6.43333	-10.7833	Wang et al. (2014)	SRP038750	SRR1206503
Oryza glaberrima	TOG5949	Africa Rice Center	Nigeria	7.5	9.06667	Wang et al. (2014)	SRP038750	SRR1206504
Oryza glaberrima	TOG7025	Africa Rice Center	Sierra Leone	9.85	-11.3167	Wang et al. (2014)	SRP038750	SRR1206505
Oryza glaberrima	TOG7102	Africa Rice Center	Mali	13.98333	-5.61639	Wang et al. (2014)	SRP038750	SRR1206506
Oryza glaberrima	TOG6203	Africa Rice Center	Guinea	10.38333	-9.3	Meyer et al. (2016)	SRP071857	SRR3231749
Oryza glaberrima	TOG7135	Africa Rice Center	Senegal	12.65	-15.4667	Meyer et al. (2016)	SRP071857	SRR3231750
Oryza glaberrima	TOG7197	Africa Rice Center	Cote d'Ivoire	7.4	-7.55	Meyer et al. (2016)	SRP071857	SRR3231751
Oryza glaberrima	TVA6749	Naturalis Biodiversity Center	Suriname	5.923333	-55.54	Van Andel et al. (2016)	SRP182896	SRR8506411

Species	Accession number	Collection	Country	Cluster	Coverage	Source publication	SRA study	SRA run
Oryza barthii	IRGC100122	International Rice Research Institute	Gambia	OB-V	high	Wang et al. (2014)	SRP037996	SRR1206362
Oryza barthii	IRGC100921	International Rice Research Institute	Unknown	OB-V	low	Wang et al. (2014)	SRP037996	SRR1206381
Oryza barthii	IRGC100922	International Rice Research Institute	Unknown	OB-IV	low	Wang et al. (2014)	SRP037996	SRR1206382
Oryza barthii	IRGC100927	International Rice Research Institute	Sierra Leone	OB-IV	low	Wang et al. (2014)	SRP037996	SRR1206383
Oryza barthii	IRGC100931	International Rice Research Institute	Mali	OB-I	high	Wang et al. (2014)	SRP037996	SRR1206363
Oryza barthii	IRGC100934	International Rice Research Institute	Mali	OB-V	high	Wang et al. (2014)	SRP037996	SRR1206364
Oryza barthii	IRGC100939	International Rice Research Institute	Unknown	OB-V	low	Wang et al. (2014)	SRP037996	SRR1206384
Oryza barthii	IRGC101240	International Rice Research Institute	Mali	OB-I	low	Wang et al. (2014)	SRP037996	SRR1206385
Oryza barthii	IRGC101252	International Rice Research Institute	Burkina Faso	OB-V	low	Wang et al. (2014)	SRP037996	SRR1206386
Oryza barthii	IRGC101381	International Rice Research Institute	Niger	OB-V	low	Wang et al. (2014)	SRP037996	SRR1206387
Oryza barthii	IRGC101959	International Rice Research Institute	Senegal	OB-V	low	Wang et al. (2014)	SRP037996	SRR1206388
Oryza barthii	IRGC103534	International Rice Research Institute	Mali	OB-I	low	Wang et al. (2014)	SRP037996	SRR1206389
Oryza barthii	IRGC103895	International Rice Research Institute	Senegal	OB-V	high	Wang et al. (2014)	SRP037996	SRR1206365
Oryza barthii	IRGC103912	International Rice Research Institute	Tanzania	OB-II	high	Wang et al. (2014)	SRP037996	SRR1206370
Oryza barthii	IRGC104084	International Rice Research Institute	Nigeria	OB-V	high	Wang et al. (2014)	SRP037996	SRR1206366
Oryza barthii	IRGC104119	International Rice Research Institute	Chad	OB-II	high	Wang et al. (2014)	SRP037996	SRR1206367
Oryza barthii	IRGC105608	International Rice Research Institute	Cameroon	OB-II	high	Wang et al. (2014)	SRP037996	SRR1206368
Oryza barthii	IRGC106234	International Rice Research Institute	Sierra Leone	OB-IV	high	Wang et al. (2014)	SRP037996	SRR1206369
Oryza barthii	WAB0009239	Africa Rice Center	Nigeria	OB-III	low	Wang et al. (2014)	SRP037996	SRR1206390
Oryza barthii	WAB0009240	Africa Rice Center	Cameroon	OB-V	low	Wang et al. (2014)	SRP037996	SRR1206391
Oryza barthii	WAB0012712	Africa Rice Center	Mali	OB-V	low	Wang et al. (2014)	SRP037996	SRR1206392
Oryza barthii	WAB0024904	Africa Rice Center	Nigeria	OB-V	low	Wang et al. (2014)	SRP037996	SRR1206393
Oryza barthii	WAB0026768	Africa Rice Center	Mali	OB-V	low	Wang et al. (2014)	SRP037996	SRR1206394
Oryza barthii	WAB0026769	Africa Rice Center	Chad	OB-V	low	Wang et al. (2014)	SRP037996	SRR1206395
Oryza barthii	WAB0026770	Africa Rice Center	Nigeria	OB-V	low	Wang et al. (2014)	SRP037996	SRR1206396
Oryza barthii	WAB0028874	Africa Rice Center	Gambia	OB-V	low	Wang et al. (2014)	SRP037996	SRR1206397
Oryza barthii	WAB0028875	Africa Rice Center	Mali	OB-I	low	Wang et al. (2014)	SRP037996	SRR1206398
Oryza barthii	WAB0028876	Africa Rice Center	Guinea	OB-V	low	Wang et al. (2014)	SRP037996	SRR1206409
Oryza barthii	WAB0028877	Africa Rice Center	Niger	OB-II	low	Wang et al. (2014)	SRP037996	SRR1206400

Species	Accession number	Collection	Country	Cluster	Coverage	Source publication	SRA study	SRA run
Oryza barthii	WAB0028882	Africa Rice Center	Cameroon	OB-II	low	Wang et al. (2014)	SRP037996	SRR1206401
Oryza barthii	WAB0028884	Africa Rice Center	Cameroon	OB-II	low	Wang et al. (2014)	SRP037996	SRR1206402
Oryza barthii	WAB0028885	Africa Rice Center	Mali	OB-V	low	Wang et al. (2014)	SRP037996	SRR1206403
Oryza barthii	WAB0028887	Africa Rice Center	Tanzania	OB-II	low	Wang et al. (2014)	SRP037996	SRR1206404
Oryza barthii	WAB0028889	Africa Rice Center	Guinea	OB-V	low	Wang et al. (2014)	SRP037996	SRR1206405
Oryza barthii	WAB0028893	Africa Rice Center	Mali	OB-IV	low	Wang et al. (2014)	SRP037996	SRR1206406
Oryza barthii	WAB0028894	Africa Rice Center	Mali	OB-IV	low	Wang et al. (2014)	SRP037996	SRR1206407
Oryza barthii	WAB0028896	Africa Rice Center	Mali	OB-V	low	Wang et al. (2014)	SRP037996	SRR1206408
Oryza barthii	WAB0028897	Africa Rice Center	Mali	OB-I	low	Wang et al. (2014)	SRP037996	SRR1206409
Oryza barthii	WAB0028900	Africa Rice Center	Mali	OB-IV	low	Wang et al. (2014)	SRP037996	SRR1206410
Oryza barthii	WAB0028903	Africa Rice Center	Zambia	OB-II	high	Wang et al. (2014)	SRP037996	SRR1206371
Oryza barthii	WAB0028905	Africa Rice Center	Senegal	OB-V	low	Wang et al. (2014)	SRP037996	SRR1206411
Oryza barthii	WAB0028907	Africa Rice Center	Senegal	OB-V	low	Wang et al. (2014)	SRP037996	SRR1206412
Oryza barthii	WAB0028910	Africa Rice Center	Mali	OB-IV	low	Wang et al. (2014)	SRP037996	SRR1206413
Oryza barthii	WAB0028911	Africa Rice Center	Mali	OB-IV	low	Wang et al. (2014)	SRP037996	SRR1206414
Oryza barthii	WAB0028912	Africa Rice Center	Mali	OB-IV	low	Wang et al. (2014)	SRP037996	SRR1206415
Oryza barthii	WAB0028913	Africa Rice Center	Mali	OB-IV	low	Wang et al. (2014)	SRP037996	SRR1206416
Oryza barthii	WAB0028915	Africa Rice Center	Mali	OB-IV	low	Wang et al. (2014)	SRP037996	SRR1206417
Oryza barthii	WAB0028916	Africa Rice Center	Mali	OB-I	low	Wang et al. (2014)	SRP037996	SRR1206418
Oryza barthii	WAB0028917	Africa Rice Center	Chad	OB-II	low	Wang et al. (2014)	SRP037996	SRR1206419
Oryza barthii	WAB0028919	Africa Rice Center	Chad	OB-V	low	Wang et al. (2014)	SRP037996	SRR1206420
Oryza barthii	WAB0028925	Africa Rice Center	Chad	OB-II	low	Wang et al. (2014)	SRP037996	SRR1206421
Oryza barthii	WAB0028926	Africa Rice Center	Chad	OB-II	low	Wang et al. (2014)	SRP037996	SRR1206422
Oryza barthii	WAB0028927	Africa Rice Center	Chad	OB-III	low	Wang et al. (2014)	SRP037996	SRR1206423
Oryza barthii	WAB0028929	Africa Rice Center	Chad	OB-II	low	Wang et al. (2014)	SRP037996	SRR1206424
Oryza barthii	WAB0028930	Africa Rice Center	Chad	OB-II	low	Wang et al. (2014)	SRP037996	SRR1206425
Oryza barthii	WAB0028931	Africa Rice Center	Chad	OB-I	low	Wang et al. (2014)	SRP037996	SRR1206426
Oryza barthii	WAB0028934	Africa Rice Center	Chad	OB-II	low	Wang et al. (2014)	SRP037996	SRR1206427
Oryza barthii	WAB0028934	Africa Rice Center	Chad	OB-II	low	Wang et al. (2014)	SRP037996	SRR1206427

Species	Accession number	Collection	Country	Cluster	Coverage	Source publication	SRA study	SRA run
Oryza barthii	WAB0028937	Africa Rice Center	Nigeria	OB-III	low	Wang et al. (2014)	SRP037996	SRR1206428
Oryza barthii	WAB0028938	Africa Rice Center	Nigeria	OB-III	high	Wang et al. (2014)	SRP037996	SRR1206429
Oryza barthii	WAB0028940	Africa Rice Center	Nigeria	OB-III	low	Wang et al. (2014)	SRP037996	SRR1206429
Oryza barthii	WAB0028942	Africa Rice Center	Cameroon	OB-II	low	Wang et al. (2014)	SRP037996	SRR1206430
Oryza barthii	WAB0028944	Africa Rice Center	Cameroon	OB-II	low	Wang et al. (2014)	SRP037996	SRR1206431
Oryza barthii	WAB0028946	Africa Rice Center	Cameroon	OB-I	low	Wang et al. (2014)	SRP037996	SRR1206432
Oryza barthii	WAB0028947	Africa Rice Center	Cameroon	OB-V	low	Wang et al. (2014)	SRP037996	SRR1206433
Oryza barthii	WAB0028948	Africa Rice Center	Cameroon	OB-V	low	Wang et al. (2014)	SRP037996	SRR1206434
Oryza barthii	WAB0028952	Africa Rice Center	Zambia	OB-III	high	Wang et al. (2014)	SRP037996	SRR1206373
Oryza barthii	WAB0028956	Africa Rice Center	Guinea	OB-V	low	Wang et al. (2014)	SRP037996	SRR1206435
Oryza barthii	WAB0028957	Africa Rice Center	Guinea	OB-V	low	Wang et al. (2014)	SRP037996	SRR1206436
Oryza barthii	WAB0028958	Africa Rice Center	Mali	OB-II	high	Wang et al. (2014)	SRP037996	SRR1206374
Oryza barthii	WAB0028959	Africa Rice Center	Mali	OB-V	low	Wang et al. (2014)	SRP037996	SRR1206437
Oryza barthii	WAB0028961	Africa Rice Center	Mali	OB-V	low	Wang et al. (2014)	SRP037996	SRR1206438
Oryza barthii	WAB0028967	Africa Rice Center	Gambia	OB-V	low	Wang et al. (2014)	SRP037996	SRR1206439
Oryza barthii	WAB0028972	Africa Rice Center	Gambia	OB-V	low	Wang et al. (2014)	SRP037996	SRR1206440
Oryza barthii	WAB0028975	Africa Rice Center	Mali	OB-I	low	Wang et al. (2014)	SRP037996	SRR1206441
Oryza barthii	WAB0028976	Africa Rice Center	Mali	OB-I	high	Wang et al. (2014)	SRP037996	SRR1206375
Oryza barthii	WAB0028977	Africa Rice Center	Mali	OB-I	low	Wang et al. (2014)	SRP037996	SRR1206442
Oryza barthii	WAB0028979	Africa Rice Center	Mali	OB-I	high	Wang et al. (2014)	SRP037996	SRR1206376
Oryza barthii	WAB0028980	Africa Rice Center	Mali	OB-I	high	Wang et al. (2014)	SRP037996	SRR1206377
Oryza barthii	WAB0028981	Africa Rice Center	Mali	OB-I	low	Wang et al. (2014)	SRP037996	SRR1206443
Oryza barthii	WAB0028983	Africa Rice Center	Cameroon	OB-II	low	Wang et al. (2014)	SRP037996	SRR1206444
Oryza barthii	WAB0028985	Africa Rice Center	Chad	OB-II	low	Wang et al. (2014)	SRP037996	SRR1206445
Oryza barthii	WAB0028987	Africa Rice Center	Nigeria	OB-IV	high	Wang et al. (2014)	SRP037996	SRR1206378
Oryza barthii	WAB0028989	Africa Rice Center	Chad	OB-II	low	Wang et al. (2014)	SRP037996	SRR1206446
Oryza barthii	WAB0028991	Africa Rice Center	Chad	OB-II	low	Wang et al. (2014)	SRP037996	SRR1206447
Oryza barthii	WAB0028992	Africa Rice Center	Chad	OB-III	high	Wang et al. (2014)	SRP037996	SRR1206379
Oryza barthii	WAB0028993	Africa Rice Center	Cameroon	OB-II	low	Wang et al. (2014)	SRP037996	SRR1206448

Species	Accession number	Collection	Country	Cluster	Coverage	Source publication	SRA study	SRA run
Oryza barthii	WAB0028994	Africa Rice Center	Nigeria	OB-II	low	Wang et al. (2014)	SRP037996	SRR1206449
Oryza barthii	WAB0028996	Africa Rice Center	Nigeria	OB-V	low	Wang et al. (2014)	SRP037996	SRR1206450
Oryza barthii	WAB0028997	Africa Rice Center	Nigeria	OB-V	low	Wang et al. (2014)	SRP037996	SRR1206451
Oryza barthii	WAB0028998	Africa Rice Center	Chad	OB-V	low	Wang et al. (2014)	SRP037996	SRR1206452
Oryza barthii	WAB0029000	Africa Rice Center	Botswana	OB-II	low	Wang et al. (2014)	SRP037996	SRR1206453
Oryza barthii	WAB0030151	Africa Rice Center	Chad	OB-IV	high	Wang et al. (2014)	SRP037996	SRR1206380
Oryza barthii	WAB0030173	Africa Rice Center	Unknown	OB-V	low	Wang et al. (2014)	SRP037996	SRR1206454
Oryza barthii	WAB0030186	Africa Rice Center	Mali	OB-IV	low	Wang et al. (2014)	SRP037996	SRR1206455

References

1. Wang M, Yu Y, Haberer G, Marri PR, Fan C, Goicoechea JL, et al. The genome sequence of African rice (Oryza glaberrima) and evidence for independent domestication. Nat Genet. 2014 Sep 27;46(9):982–8.

2. Meyer RS, Choi JY, Sanches M, Plessis A, Flowers JM, Amas J, et al. Domestication history and geographical adaptation inferred from a SNP map of African rice. Nat Genet. 2016 Aug 8;48(9):1083–8.

3. Van Andel TR, Meyer RS, Aflitos SA, Carney JA, Veltman MA, Copetti D, et al. Tracing ancestor rice of Suriname Maroons back to its African origin. Nat Plants. 2016 Oct 3;2(10):16149.
