# Supplementary material for: Origins and geographic diversification of African rice (Oryza glaberrima)
Source: PLoS One. 2019 Mar 6;14(3):e0203508. doi: 10.1371/journal.pone.0203508 (PMC6402627; doi:10.1371/journal.pone.0203508)
Supplement: S2 Table — (PDF) [file pone.0203508.s002.pdf]

S2 Table. Genes selected for phylogenetic analysis. Domestication stage is only given for genes taken from Li et al. [1], because this was not reported by Wang et al. [2]. The Rice Annotation Project Database (RAP-DB) utilises the *Oryza sativa* ssp. japonica cv. Nipponbare genome and is accessible on http://rapdb.dna.affrc.go.jp/ [3].

**Homologues of known domestication genes in *Oryza sativa***				**RAP-DB query results (*Oryza sativa*)**		**BLAST+ query results (*Oryza glaberrima*)**		**Sequence identity**
**Gene**	**Domestication stage**	**Function**	**Source**	**ID**	**Genomic coordinates**	**ID**	**Genomic coordinates**	
**OsLG1**	Domestication	Closed panicle	Li et al. (2017) & Wang et al. (2014)	Os04g0656500	chr04:33488722:33492700	ORGLA04G0242500	chr04:24455085:24458509	99%
**Sh4**	Domestication	Seed shattering	Li et al. (2017) & Wang et al. (2014)	Os04g0670900	chr04:34231186:34233221	ORGLA04G0254300	chr04:25150788:25152622	98%
**qSh1**	Early improvement	Seed shattering	Li et al. (2017) & Wang et al. (2014)	Os01g0848400	chr01:36445456:36449951	ORGLA01G0311100	chr01:26836716:26840603	99%
**Bh4**	Early improvement	Hull colour	Li et al. (2017)	Os04g0460200	chr04:22969845:22971859	ORGLA04G0119300	chr04:15433128:15433925	99%
**LABA1**	Early improvement	Awn barb	Li et al. (2017)	Os04g0518800	chr04:25959399:25963504	ORGLA04G0160000	chr04:18237532:18241208	100%
**OsC1**	Early improvement	Hull colour	Li et al. (2017)	Os06g0205100	chr06:5315163:5316640	ORGLA06G0059500	chr06:4466255:4467544	98%
**Phr1**	Later improvement	Grain discoloration	Li et al. (2017) & Wang et al. (2014)	Os04g0624500	chr04:31749141:31751604	ORGLA04G0229000	chr04:23491785:23494110	93%
**COLD1**	Later improvement	Chilling tolerance	Li et al. (2017)	Os04g0600800	chr04:30311574:30316221	ORGLA04G0206300	chr04:21690765:21694785	99%
**Waxy**	Later improvement	Amylose content	Li et al. (2017)	Os06g0133000	chr06:1765622:1770653	ORGLA06G0020500	chr06:1582446:1585771	99%
**Rc**	Later improvement	Red pericarp	Li et al. (2017) & Wang et al. (2014)	Os07g0211500	chr07:6062889:6069304	ORGLA07G0060000	chr07:5268358:5270384	98%
**Sd1**		Semi-dwarfing	Wang et al. (2014)	Os01g0883800	chr01:38382385:38385469	ORGLA01G0334700	chr01:28541899:28544581	98%
**Gn1a**		Grain productivity	Wang et al. (2014)	Os01g0197700	chr01:5270449:5275585	ORGLA01G0059300	chr01:4236446:4240917	98%
**GW2**		Grain width	Wang et al. (2014)	Os02g0244100	chr02:8115223:8121651	ORGLA02G0093000	chr02:7561612:7567493	99%
**GIF1**		Grain incomplete filling	Wang et al. (2014)	Os04g0413500	chr04:20422171:20426921	ORGLA04G0089800	chr04:13126506:13130922	99%
**MOC1**		Tillering	Wang et al. (2014)	Os06g0610350	chr06:24313523:24316697	ORGLA06G0172100	chr06:17930149:17931657	99%
**Sdr4**		Seed dormancy	Wang et al. (2014)	Os07g0585700	chr07:23796611:23797642	ORGLA07G0155600	chr07:16905004:16906038	97%
**Ep2**		Erect panicle	Wang et al. (2014)	Os07g0616000	chr07:25381698:25389532	ORGLA07G0170500	chr07:18073478:18080578	99%
**Badh2**		Fragrance	Wang et al. (2014)	Os08g0424500	chr08:20379823:20385975	ORGLA08G0132000	chr08:15046659:15052508	100%
**IPA1**		Ideal plant architure	Wang et al. (2014)	Os08g0509600	chr08:25274541:25278696	ORGLA08G0176500	chr08:19078252:19082033	99%
**Dep1**		Dense and erect panicle	Wang et al. (2014)	Os09g0441900	chr09:16411151:16415851	ORGLA09G0089300	chr09:12041569:12045716	99%
Prog1	*Domestication*	*Plant architecture*	*Li et al. (2017) & Wang et al. (2014)*	*Os07g0153600*	*chr07:2839194:2840089*	*Not found*	*?*	
An-1	*Early improvement*	*Awn length*	*Li et al. (2017)*	*Os04g0350700*	*chr04:16731738:16735336*	*Not found*	*?*	
GS3		*Grain size and weight*	*Wang et al. (2014)*	*Os03g0407400*	*chr03:16729501:16735109*	*Not found*	*?*	
OsSh1		*Seed shattering*	*Wang et al. (2014)*	*Os03g0650000*	*chr03:25197057:25206948*	*Not found*	*Deleted*	
Hd1		*Heading date*	*Wang et al. (2014)*	*Os06g0275000*	*chr06:9336376:9338569*	*Not found*	*Deleted*	

References

1. Li L-F, Li Y-L, Jia Y, Caicedo AL, Olsen KM. Signatures of adaptation in the weedy rice genome. Nat Genet. 2017 Apr 3;49(5):811–4.
2. Wang M, Yu Y, Haberer G, Marri PR, Fan C, Goicoechea JL, et al. The genome sequence of African rice (Oryza glaberrima) and evidence for independent domestication. Nat Genet. 2014 Sep 27;46(9):982–8.
3. Sakai H, Lee SS, Tanaka T, Numa H, Kim J, Kawahara Y, et al. Rice Annotation Project Database (RAP-DB): An Integrative and Interactive Database for Rice Genomics. Plant Cell Physiol. 2013 Feb;54(2):e6–e6.
