# Supplementary material for: Origins and geographic diversification of African rice (Oryza glaberrima)
Source: PLoS One. 2019 Mar 6;14(3):e0203508. doi: 10.1371/journal.pone.0203508 (PMC6402627; doi:10.1371/journal.pone.0203508)
Supplement: S3 Table — (PDF) [file pone.0203508.s003.pdf]

S3 Table. Phasing and phylogenetic output of domestication genes. Data are based on genomic intervals spanning the gene, plus 5 kb on either side. Columns show the number of loci and the number of haplotypes per gene; the frequency of the most prevalent haplotype; and the number of leaves and sum of branch lengths (SBL) of the corresponding NJ tree. Bordered rows indicate genes in which one or more of the five largest haplotypes are composed of *O. glaberrima* individuals belonging to a single population, namely OG-II (**red**), or OG-IV (**pink**). NJ trees of these genes can be found in Fig 6 of the main text and in Fig S7 of the Supplementary information.

**Gene**	**Chrom**	**Start**	**End**	**Loci**	**Haplotypes**	**Highest frequency**	**Leaves**	**SBL**
**qSh1**	1	26831714	26845603	91	63	0.55	412	2.63252201
**Sd1**	1	28536897	28549581	136	101	0.47	412	3.46575573
**Gn1a**	1	4231445	4245917	2	4	0.82	412	1.25
**Gw2**	2	7556611	7572493	207	92	0.4	412	2.59930774
**Gif1**	4	13121505	13135922	190	264	0.12	412	7.3951465
**Bh4**	4	15428126	15438925	119	72	0.65	412	2.18847926
**LABA1**	4	18232530	18246208	245	144	0.2	412	2.49139267
**COLD1**	4	21685763	21699785	63	37	0.42	412	1.84506179
**Phr1**	4	23486784	23499110	113	53	0.5	412	1.57358009
**OsLG1**	4	24450083	24463509	119	75	0.51	412	2.41602114
**Sh4**	4	25145786	25157622	131	102	0.29	412	2.6869362
**Waxy**	6	1577444	1590771	266	110	0.3	412	2.74459838
**MOC1**	6	17925148	17936657	96	67	0.58	412	2.64005463
**OsC1**	6	4461253	4472544	200	97	0.21	412	2.22526556
**Sdr4**	7	16900003	16911038	153	86	0.57	412	3.5857042
**Rc**	7	5263356	5275384	84	65	0.52	412	2.11471613
**EP2**	7	18068476	18085578	151	200	0.27	412	5.17523044
**Badh2**	8	15041658	15057508	114	96	0.5	412	2.4141847
**IPA1**	8	19073251	19087033	119	47	0.63	412	1.59925443
**Dep1**	9	12036567	12050716	241	119	0.42	412	2.73050577
**Average**				**142**	**95**	**0.45**	**412**	**2.79**
