# Supplementary material for: Origins and geographic diversification of African rice (Oryza glaberrima)
Source: PLoS One. 2019 Mar 6;14(3):e0203508. doi: 10.1371/journal.pone.0203508 (PMC6402627; doi:10.1371/journal.pone.0203508)
Supplement: S4 Table — (PDF) [file pone.0203508.s004.pdf]

S4 Table. Description of quality control filters. All filter recommendations were taken from GATK Best Practices [1], with the exception of 'Missing data'.

Filter	Meaning	Rationale	Remark
**DP**	Depth of Coverage	Removes sites with excessive coverage caused by alignment artefacts.	Sequencing depth of more than 5 or 6 sigma from the mean depth is extremely unlikely given a Gaussian distribution of read depth.
**QD**	Quality by Depth	Removes sites with low confidence (variant quality divided by depth).	The Phred quality score is normalised to avoid inflation caused by deep sequencing.
**MQ**	RMS Mapping Quality	Removes sites with a low Root Mean Square (RMS) mapping quality.	The root of the mean square is taken to take account for variability in the dataset.
**MQRankSum**	Mapping Quality Rank Sum Test	Removes sites where the mapping qualities of the reference and alternate alleles are not comparable.	The u-based z-approximation compares the mapping qualities of the reads supporting the reference allele and the alternate allele.
**ReadPosRankSum**	Read Position Rank Sum Test	Removes sites where one allele occurs more frequently at the end of reads than the other, because this is where sequencing errors occur.	The u-based z-approximation compares whether the positions of the reference and alternate alleles are different within the reads.
**FS**	Strand Bias (Fisher's exact test)	Removes sites where the alternate allele was seen more or less often on the forward or reverse strand than the reference allele.	The probability of strand bias is Phred-scaled.
**Missing data**	Percentage of missing genotypes	Removes sites where the call rate is low.	The number of genotypes that are not called is divided by the total number of genotypes.

References

1. Broad Institute. GATK | Best Practices [Internet]. [cited 2017 Aug 7]. Available from: https://software.broadinstitute.org/gatk/best-practices/
