# Supplementary material for: Origins and geographic diversification of African rice (Oryza glaberrima)
Source: PLoS One. 2019 Mar 6;14(3):e0203508. doi: 10.1371/journal.pone.0203508 (PMC6402627; doi:10.1371/journal.pone.0203508)
Supplement: S5 Table — (PDF) [file pone.0203508.s005.pdf]

S5 Table. Quality control filter thresholds. Parameters were set to improve the Ts:Tv ratio while retaining a sufficient number of SNPs. Both lenient and stringent thresholds were applied when multiple cut-offs seemed justified, resulting in different versions of the same call set (call set 1a and 1b).

**Call set**	**Filter thresholds**							**Before filtering**		**After filtering**	
	**DP**	**QD**	**MQ**	**MQRankSum**	**ReadPosRankSum**	**FS**	**Missing data**	**SNP count**	**Ts:Tv ratio**	**SNP count**	**Ts:Tv ratio**
1a (lenient)	> 4800	<2	<38	<-5	<-2.5, >2.5	>60	>25%	10759580	2.44	3923601	2.57
1b (stringent)	>4800	<21	<38	<-5	<-2.5, >2.5	>30	>25%	10759580	2.44	2644126	2.65
