# Supplementary material for: Origins and geographic diversification of African rice (Oryza glaberrima)
Source: PLoS One. 2019 Mar 6;14(3):e0203508. doi: 10.1371/journal.pone.0203508 (PMC6402627; doi:10.1371/journal.pone.0203508)
Supplement: S6 Table — (PDF) [file pone.0203508.s006.pdf]

S6 Table. Summary of variant calls. Note: The relatively equal heterozygosity levels in call set 1 are predominantly caused by the comparatively low coverage *O. barthii* accessions. When removing excessively low coverage accessions (<4X) or removing lower quality SNPs (QD<21), *O. barthii* is shown to be more heterozygous than *O. glaberrima* as expected.

**Call set**	**1a**			**1b**		
Population	**Total**	***O. barthii***	***O. glaberrima***	**Total**	***O. barthii***	***O. glaberrima***
Accessions	206	94	112	206	94	112
SNP count	3,923,601	3,797,182	2,322,659	2,644,126	2,580,362	1,419,601
Unique	1,727,361	1,600,942	126,419	1,288,289	1224525	63764
Shared	2,196,240	58%	95%	1,355,837	53%	96%
Singletons	239,322	345,025	597,486	116,390	151,804	456,772
Transitions	2,823,188	2,733,322	1,686,367	1,919,367	1,872,667	1,043,405
Transversions	1,100,413	1,063,860	636,292	724,759	707,695	376,196
Ratio (Ts:Tv)	2.57	2.57	2.65	2.65	2.65	2.77
Protein coding		288,061	164,001		188,196	97,532
Coding fraction		0.076	0.071		0.073	0.069
Synonymous		130,054	73,962		84,662	42,890
Non-synonymous		153,612	87,468		100,894	53,136
Ratio (dS/dN)		0.847	0.846		0.839	0.807
Depth of coverage	11.26	5.58	16.03	10.5	5.28	14.87
Fraction missing data	0.08	0.15	0.02	0.08	0.16	0.01
Heterozygosity (all)	4.95%	4.67%	5.19%	1.59%	2.49%	0.84%
Heterozygosity (>4X)	6.15%	9.29%	5.33%	1.91%	6.08%	0.87%
