# Supplementary material for: Origins and geographic diversification of African rice (Oryza glaberrima)
Source: PLoS One. 2019 Mar 6;14(3):e0203508. doi: 10.1371/journal.pone.0203508 (PMC6402627; doi:10.1371/journal.pone.0203508)
Supplement: S7 Table — (PDF) [file pone.0203508.s007.pdf]

S7 Table. Potential outgroup species. All are wild species belonging to the *Oryza* genus. Genetic distance is based on 53 nuclear genes and 16 intergenic regions [1]. Data loss was estimated from ~1000 randomly chosen SNPs.

**Species**	**Origin**	**Clade**	**Genetic distance**	**Divergence time**	**Data loss**
O. longistaminata	Africa	AA	0.0216	2.42 mya	38%
O. meridionalis	Australia	AA	0.0301	2.93 mya	66%
O. punctata	Africa	BB	0.0637	9.11 mya	79%

References

1. Zhu T, Xu P-Z, Liu J-P, Peng S, Mo X-C, Gao L-Z. Phylogenetic relationships and genome divergence among the AA- genome species of the genus Oryza as revealed by 53 nuclear genes and 16 intergenic regions. Mol Phylogenet Evol. 2014 Jan;70:348–61.
