# Supplementary material for: Origins and geographic diversification of African rice (Oryza glaberrima)
Source: PLoS One. 2019 Mar 6;14(3):e0203508. doi: 10.1371/journal.pone.0203508 (PMC6402627; doi:10.1371/journal.pone.0203508)
Supplement: S8 Table — (PDF) [file pone.0203508.s008.pdf]

S8 Table. Candidate selective sweeps unique to *O. glaberrima*. Candidate selective sweeps are defined as outlier positions with the top 0.5% highest CLR scores across the genome, based on the likelihood method developed by Kim & Nielsen [1].

Chromosome	Position	Log(CLR)	Chromosome	Position	Log(CLR)
**1**	1426959	3.237867771	**6**	22426628	3.116000663
**1**	6808194	3.136167033	**6**	22451658	3.814361598
**1**	6833223	3.620796766	**6**	24053578	3.122649421
**1**	11889081	3.689188881	**8**	1078187	3.717595158
**1**	12239487	4.560417696	**8**	1103237	4.717364514
**1**	13340763	3.408787603	**9**	7189035	3.276112502
**1**	15443199	3.331975127	**9**	7639764	3.184903195
**2**	6658489	3.415667253	**10**	1351828	3.263180095
**3**	20764430	3.299420917	**10**	1376848	3.508927986
**3**	24278996	3.778492756	**10**	1451911	3.248242104
**3**	24303922	3.130393009	**10**	9808814	5.146302872
**4**	3179772	3.216416884	**10**	9833835	5.01910125
**4**	9013383	3.253914691	**10**	9883877	4.164606657
**4**	12067897	3.802813514	**10**	13111586	3.699459339
**4**	12318267	3.255005304	**10**	13161628	3.333576247
**4**	17350682	3.149078873	**10**	16814692	4.179178298
**5**	6211752	3.409361167	**10**	16839712	3.812071059
**5**	22015778	3.410409784	**10**	16864732	3.630679045
**6**	801346	5.080582611	**11**	12547320	8.685175038
**6**	826375	4.94629044	**11**	16779756	3.3205646
**6**	951520	3.580609711	**11**	16804800	3.238301087
**6**	9311207	3.423120602	**11**	16829844	3.473514074
**6**	9361265	3.500493033	**11**	18257352	3.661359608
**6**	11814107	4.601525322	**12**	12223437	3.485106635
**6**	11839136	4.601525322	**12**	12248446	3.357935155
**6**	16669733	3.377263266	**12**	12273455	3.317696418
**6**	16694762	3.516827286	**12**	12298464	3.789117065
**6**	21250218	3.254181152	**12**	12648590	3.268978613

We identified 1120 putative moderate to high impact mutations in 278 genes located less than 25 kb away from candidate sweeps. Of these mutations, 34 had a severe impact and were located in 17 different genes. These genes, their predicted function and the nature of their associated high impact mutations can be found in S18 Table and S19 Table, respectively.

References

1. Kim Y, Nielsen R. Linkage Disequilibrium as a Signature of Selective Sweeps. Genetics. 2004;167(3).
