# Supplementary material for: Origins and geographic diversification of African rice (Oryza glaberrima)
Source: PLoS One. 2019 Mar 6;14(3):e0203508. doi: 10.1371/journal.pone.0203508 (PMC6402627; doi:10.1371/journal.pone.0203508)
Supplement: S9 Table — (PDF) [file pone.0203508.s009.pdf]

S9 Table. Genes associated with candidate sweeps. Gene functions are based on ontology (GO) terms and available through UniProtKB [1].

ID	Chrom	Start	End	Strand	Description	GO category	Function	Activity
ORGLA01G0020300	1	1421693	1424058	-	Uncharacterised	Molecular function	ATP binding	protein serine/threonine kinase activity
ORGLA01G0020500	1	1430691	1431574	+	Uncharacterised	NA	NA	NA
ORGLA01G0020500	1	1430691	1431574	+	Uncharacterised	NA	NA	NA
ORGLA01G0020500	1	1430691	1431574	+	Uncharacterised	NA	NA	NA
ORGLA02G0085500	2	6652855	6655870	-	Auxin responsive protein	Biological process	auxin-activated signaling pathway	regulation of transcription, DNA-templated
ORGLA02G0085500	2	6652855	6655870	-	Auxin responsive protein	Biological process	auxin-activated signaling pathway	regulation of transcription, DNA-templated
ORGLA05G0069500	5	6221541	6224335	-	Uncharacterised	NA	NA	NA
ORGLA05G0069600	5	6230822	6232728	-	Uncharacterised	NA	NA	NA
ORGLA05G0069600	5	6230822	6232728	-	Uncharacterised	NA	NA	NA
ORGLA05G0069600	5	6230822	6232728	-	Uncharacterised	NA	NA	NA
ORGLA05G0069600	5	6230822	6232728	-	Uncharacterised	NA	NA	NA
ORGLA06G0208300	6	21265153	21266448	+	Uncharacterised	NA	NA	NA
ORGLA06G0225700	6	22433573	22434991	+	Uncharacterised	NA	NA	NA
ORGLA06G0225500	6	22409271	22413960	-	Uncharacterised	Molecular function	ADP binding	NA
ORGLA06G0225500	6	22409271	22413960	-	Uncharacterised	Molecular function	ADP binding	NA
ORGLA06G0225500	6	22409271	22413960	-	Uncharacterised	Molecular function	ADP binding	NA
ORGLA06G0225500	6	22409271	22413960	-	Uncharacterised	Molecular function	ADP binding	NA
ORGLA06G0225500	6	22409271	22413960	-	Uncharacterised	Molecular function	ADP binding	NA
ORGLA06G0225700	6	22433573	22434991	+	Uncharacterised	NA	NA	NA
ORGLA09G0045900	9	7196691	7197119	+	Uncharacterised	NA	NA	NA
ORGLA09G0045900	9	7196691	7197119	+	Uncharacterised	NA	NA	NA
ORGLA09G0045900	9	7196691	7197119	+	Uncharacterised	NA	NA	NA
ORGLA09G0046000	9	7204473	7205627	-	Uncharacterised	NA	NA	NA
ORGLA09G0046000	9	7204473	7205627	-	Uncharacterised	NA	NA	NA
ORGLA10G0070100	10	9785288	9786138	+	Uncharacterised	NA	NA	NA
ORGLA10G0070100	10	9785288	9786138	+	Uncharacterised	NA	NA	NA
ORGLA10G0150600	10	16798552	16799153	-	Uncharacterised	NA	NA	NA
ORGLA11G0153900	11	16778633	16780936	-	Uncharacterised	Biological process	regulation of monopolar cell growth	NA
ORGLA11G0154200	11	16829453	16829740	+	Uncharacterised	Molecular function	ADP binding	NA
ORGLA11G0154200	11	16829453	16829740	+	Uncharacterised	Molecular function	ADP binding	NA
ORGLA11G0154300	11	16834322	16835425	+	Uncharacterised	Molecular function	ADP binding	NA
ORGLA11G0154400	11	16835730	16836384	+	Uncharacterised	NA	NA	NA
ORGLA11G0154700	11	16847944	16853200	+	Uncharacterised	Molecular function	ADP binding	NA
ORGLA11G0154700	11	16847944	16853200	+	Uncharacterised	Molecular function	ADP binding	NA

References

1. The UniProt Consortium. UniProt: the Universal Protein knowledgebase. Nucleic Acids Res. 2017 Jan 4;45(D1):D158–D169.
