# Supplementary material for: Origins and geographic diversification of African rice (Oryza glaberrima)
Source: PLoS One. 2019 Mar 6;14(3):e0203508. doi: 10.1371/journal.pone.0203508 (PMC6402627; doi:10.1371/journal.pone.0203508)
Supplement: S10 Table — (PDF) [file pone.0203508.s010.pdf]

S10 Table. High impact mutations associated with candidate sweeps. Predicted using SnpEff and SnpSift [1,2].

ID	Chrom	Start	End	Position	Reference	Alternate	Variant	Impact	Effect	Codon change	Amino acid change
**ORGLA01G0020300**	1	1421693	1424058	1421693	T	G	G	HIGH	stop_lost&splice_region_variant	c.1272A>C	p.Ter424Cysext*?
**ORGLA01G0020500**	1	1430691	1431574	1430757	C	T	T	HIGH	stop_gained	c.67C>T	p.Arg23*
**ORGLA01G0020500**	1	1430691	1431574	1430760	G	T	T	HIGH	stop_gained	c.70G>T	p.Gly24*
**ORGLA01G0020500**	1	1430691	1431574	1430889	T	C	C	HIGH	stop_lost	c.199T>C	p.Ter67Glnext*?
**ORGLA02G0085500**	2	6652855	6655870	6655658	G	T	T	HIGH	stop_gained	c.213C>A	p.Cys71*
**ORGLA02G0085500**	2	6652855	6655870	6655661	G	T	T	HIGH	stop_gained	c.210C>A	p.Cys70*
**ORGLA05G0069500**	5	6221541	6224335	6222036	C	G	G	HIGH	stop_lost	c.669G>C	p.Ter223Tyrext*?
**ORGLA05G0069600**	5	6230822	6232728	6230839	G	A	A	HIGH	stop_gained	c.544C>T	p.Arg182*
**ORGLA05G0069600**	5	6230822	6232728	6230854	G	A	A	HIGH	stop_gained	c.529C>T	p.Gln177*
**ORGLA05G0069600**	5	6230822	6232728	6231255	C	T	T	HIGH	splice_acceptor_variant&intron_variant	c.130-2G>A	
**ORGLA05G0069600**	5	6230822	6232728	6231366	G	A	A	HIGH	stop_gained	c.124C>T	p.Arg42*
**ORGLA06G0208300**	6	21265153	21266448	21265336	G	T	T	HIGH	stop_gained	c.184G>T	p.Glu62*
**ORGLA06G0225700**	6	22433573	22434991	22434989	T	C	C	HIGH	stop_lost&splice_region_variant	c.1282T>C	p.Ter428Argext*?
**ORGLA06G0225500**	6	22409271	22413960	22409393	G	A	A	HIGH	stop_gained	c.4315C>T	p.Gln1439*
**ORGLA06G0225500**	6	22409271	22413960	22410158	G	A	A	HIGH	stop_gained	c.3550C>T	p.Gln1184*
**ORGLA06G0225500**	6	22409271	22413960	22410310	T	C	C	HIGH	stop_lost	c.3398A>G	p.Ter1133Trpext*?
**ORGLA06G0225500**	6	22409271	22413960	22410991	T	C	C	HIGH	stop_lost	c.2717A>G	p.Ter906Trpext*?
**ORGLA06G0225500**	6	22409271	22413960	22411742	G	A	A	HIGH	stop_gained	c.1966C>T	p.Gln656*
**ORGLA06G0225700**	6	22433573	22434991	22434989	T	C	C	HIGH	stop_lost&splice_region_variant	c.1282T>C	p.Ter428Argext*?
**ORGLA09G0045900**	9	7196691	7197119	7196692	T	C	C	HIGH	start_lost	c.2T>C	p.Met1?
**ORGLA09G0045900**	9	7196691	7197119	7197051	C	T	T	HIGH	stop_gained	c.361C>T	p.Gln121*
**ORGLA09G0045900**	9	7196691	7197119	7197119	A	G	G	HIGH	stop_lost&splice_region_variant	c.429A>G	p.Ter143Trpext*?
**ORGLA09G0046000**	9	7204473	7205627	7204742	C	A	A	HIGH	stop_gained	c.886G>T	p.Glu296*
**ORGLA09G0046000**	9	7204473	7205627	7205263	C	T	T	HIGH	stop_gained	c.365G>A	p.Trp122*
**ORGLA10G0070100**	10	9785288	9786138	9785597	T	C	C	HIGH	stop_lost	c.310T>C	p.Ter104Argext*?
**ORGLA10G0070100**	10	9785288	9786138	9785822	A	G	G	HIGH	splice_acceptor_variant&intron_variant	c.354-1A>G	
**ORGLA10G0150600**	10	16798552	16799153	16798654	A	C	C	HIGH	splice_acceptor_variant&intron_variant	c.253-1T>G	
**ORGLA11G0153900**	11	16778633	16780936	16779436	G	A	A	HIGH	stop_gained	c.1411C>T	p.Gln471*
**ORGLA11G0154200**	11	16829453	16829740	16829528	C	T	T	HIGH	stop_gained	c.76C>T	p.Gln26*
**ORGLA11G0154200**	11	16829453	16829740	16829528	C	T	T	HIGH	stop_gained	c.76C>T	p.Gln26*
**ORGLA11G0154300**	11	16834322	16835425	16835368	T	A	A	HIGH	stop_gained	c.1047T>A	p.Tyr349*
**ORGLA11G0154400**	11	16835730	16836384	16836131	G	A	A	HIGH	stop_gained	c.227G>A	p.Trp76*
**ORGLA11G0154700**	11	16847944	16853200	16848137	G	A	A	HIGH	stop_gained	c.194G>A	p.Trp65*
**ORGLA11G0154700**	11	16847944	16853200	16851754	T	G	G	HIGH	stop_gained	c.1305T>G	p.Tyr435*

References

1. Cingolani P, Patel VM, Coon M, Nguyen T, Land SJ, Ruden DM, et al. Using Drosophila melanogaster as a model for genotoxic chemical mutational studies with a new program, SnpSift. Front Genet. 2012;3(35).

2. Cingolani P, Platts A, Wang l. L, Coon M, Nguyen T, Wang L, et al. A program for annotating and predicting the effects of single nucleotide polymorphisms, SnpEff: SNPs in the genome of Drosophila melanogaster strain w1118; iso-2; iso-3. Fly. 2012;6(2):80–92.
