# Supplementary material for: Origins and geographic diversification of African rice (Oryza glaberrima)
Source: PLoS One. 2019 Mar 6;14(3):e0203508. doi: 10.1371/journal.pone.0203508 (PMC6402627; doi:10.1371/journal.pone.0203508)
Supplement: S1 Fig — (PDF) [file pone.0203508.s011.pdf]

A B

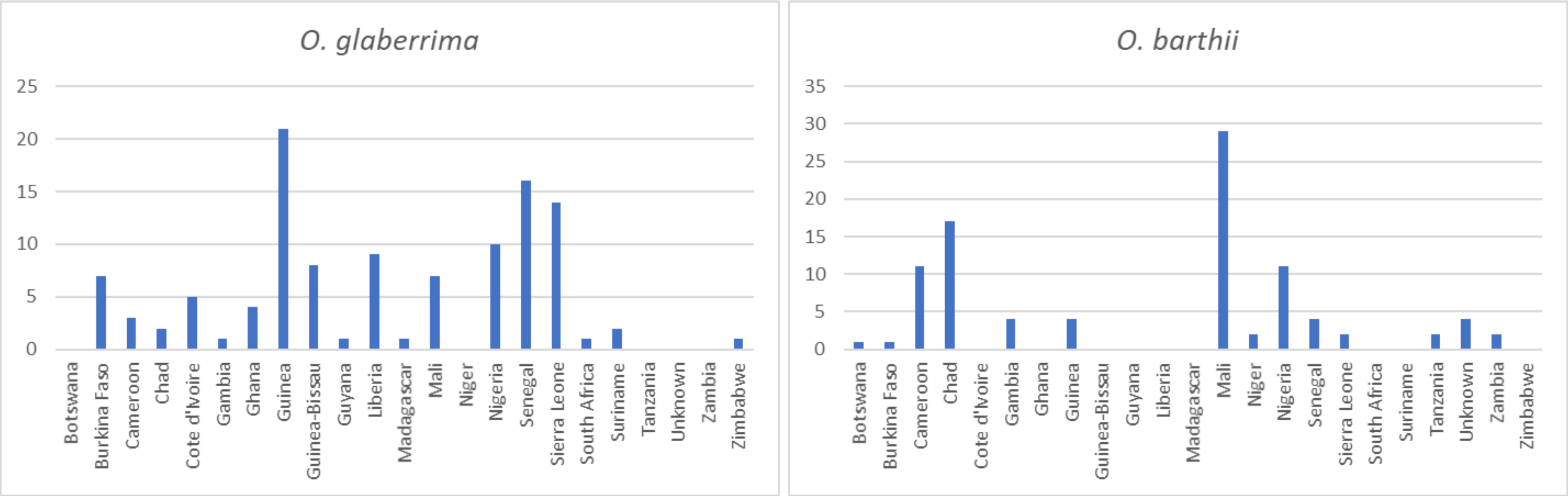

S1 Fig. Geographic origin of used accessions. A. Number of *O. glaberrima* accessions collected per country. B. Number of *O. barthii* accessions collected per country.
