# Supplementary material for: Origins and geographic diversification of African rice (Oryza glaberrima)
Source: PLoS One. 2019 Mar 6;14(3):e0203508. doi: 10.1371/journal.pone.0203508 (PMC6402627; doi:10.1371/journal.pone.0203508)
Supplement: S2 Fig — (PDF) [file pone.0203508.s012.pdf]

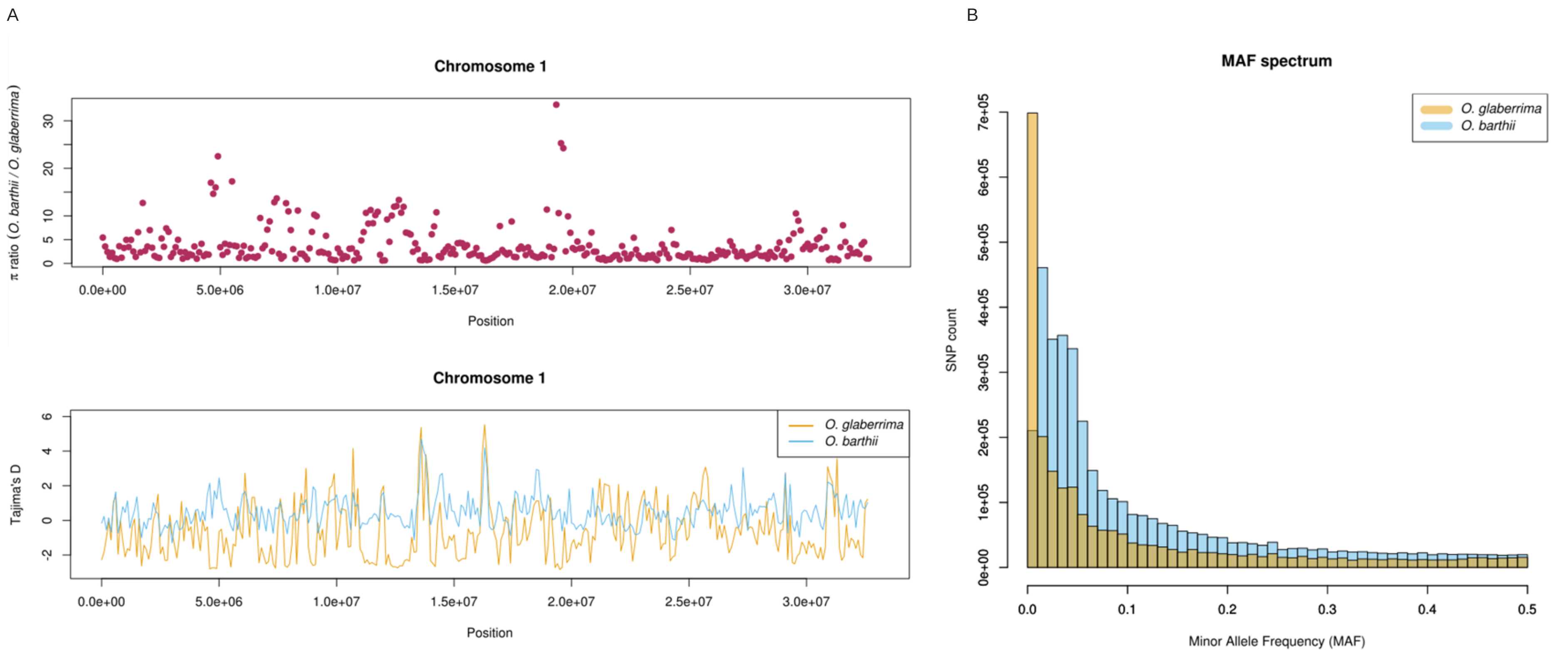

S2 Fig. Relative genetic diversity and allele frequencies in domesticated and wild rice. A. Nucleotide diversity (π) ratio and Tajima's D in *O. glaberrima* and *O. barthii* along chromosome 1, calculated in window sizes of 100 kb. Strong differences in Tajima's D correspond to outliers in π. B. Minor allele frequency spectra of *O. glaberrima* and *O. barthii*. *O. glaberrima* has an excess of low frequency variants (MAF < 0.01), whereas *O. barthii* has more intermediate frequency variants (MAF 0.01-0.05).
