# Supplementary material for: Origins and geographic diversification of African rice (Oryza glaberrima)
Source: PLoS One. 2019 Mar 6;14(3):e0203508. doi: 10.1371/journal.pone.0203508 (PMC6402627; doi:10.1371/journal.pone.0203508)
Supplement: S3 Fig — (PDF) [file pone.0203508.s013.pdf]

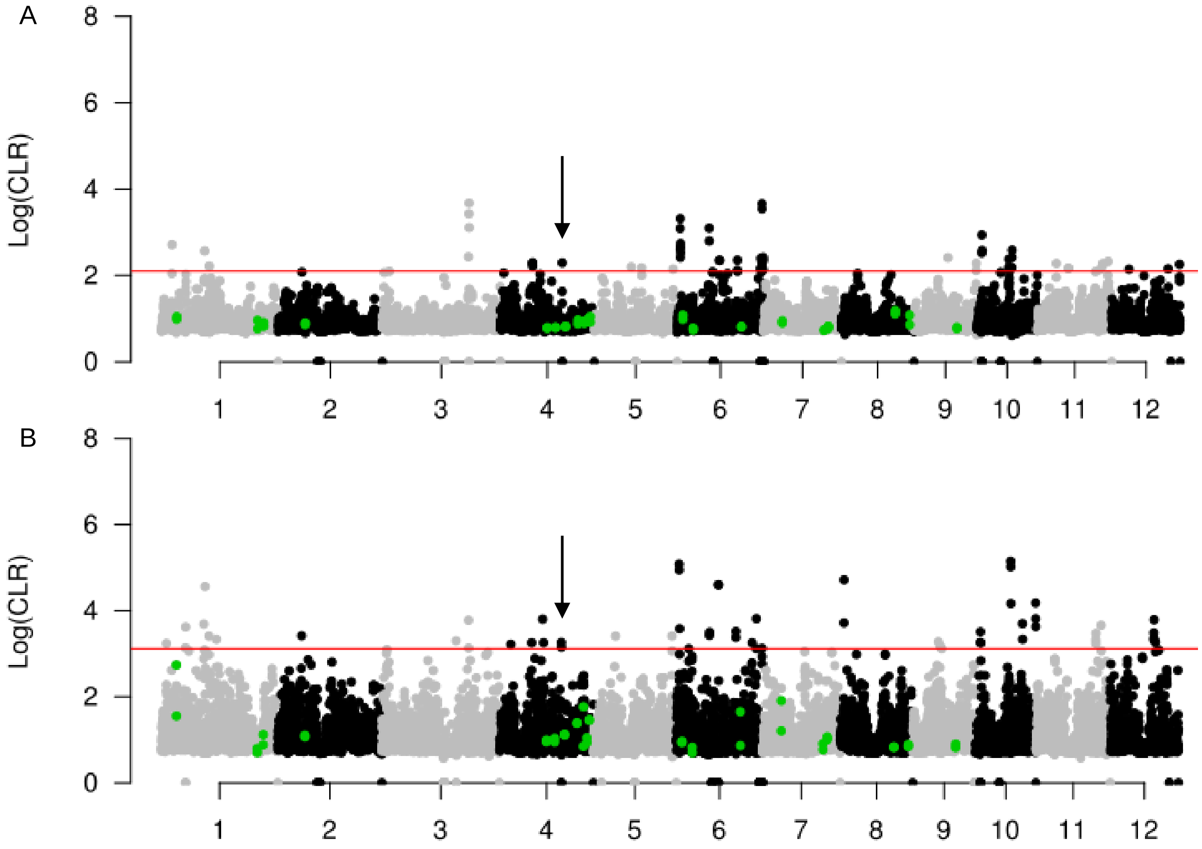

S3 Fig. Log-transformed CLR test statistic (ω), used to scan for genomic regions under selection. A. Selective sweeps in *O. barthii*. B. Selective sweeps in O. *glaberrima*. Positions less than 25kb away from a known domestication gene are highlighted in green. Positions with the top 0.5% highest values for ω are considered outlier positions and are separated by a red line. Shared outliers are indicated with a black arrow.
