# Supplementary material for: Origins and geographic diversification of African rice (Oryza glaberrima)
Source: PLoS One. 2019 Mar 6;14(3):e0203508. doi: 10.1371/journal.pone.0203508 (PMC6402627; doi:10.1371/journal.pone.0203508)
Supplement: S4 Fig — (PDF) [file pone.0203508.s014.pdf]

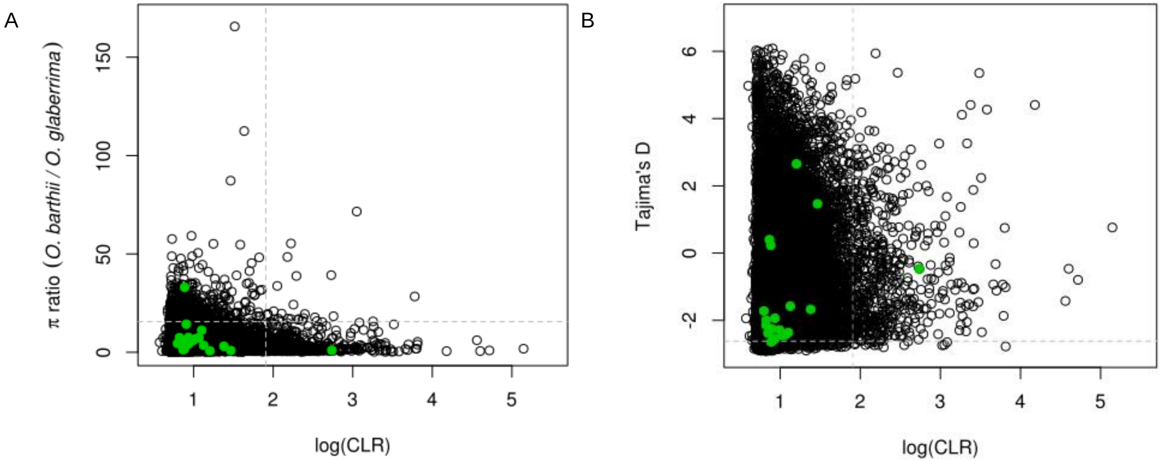

S4 Fig. Correspondence between ω and other neutrality tests. A. The ω-statistic (log(CLR)) versus relative nucleotide diversity (π ratio) in windows of 25 kb. B. The ω-statistic (log(CLR)) versus Tajima's D in windows of 25 kb. Regions harbouring domestication genes are highlighted in green. Dashed grey lines depict 5% cut-off points**.**
