# Supplementary material for: Origins and geographic diversification of African rice (Oryza glaberrima)
Source: PLoS One. 2019 Mar 6;14(3):e0203508. doi: 10.1371/journal.pone.0203508 (PMC6402627; doi:10.1371/journal.pone.0203508)
Supplement: S5 Fig — (PDF) [file pone.0203508.s015.pdf]

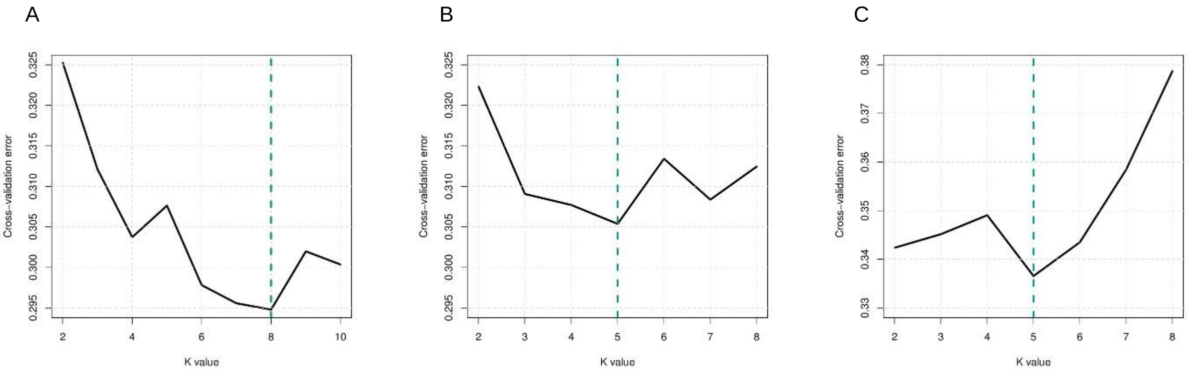

S5 Fig. Cross-validation (CV) error estimates of ADMIXTURE, with varying levels of K. A. CV error of the entire population (206 accessions), reaching a minimum at K=8. B. CV error of *O. glaberrima* (112 accessions), reaching a minimum at K=5. C. CV error of *O. barthii* (94 accessions), reaching a minimum at K=5. Bar plots of the corresponding structure analyses of A, B and C can be found in Fig 3 of the main text.
