# Supplementary material for: Origins and geographic diversification of African rice (Oryza glaberrima)
Source: PLoS One. 2019 Mar 6;14(3):e0203508. doi: 10.1371/journal.pone.0203508 (PMC6402627; doi:10.1371/journal.pone.0203508)
Supplement: S6 Fig — (PDF) [file pone.0203508.s016.pdf]

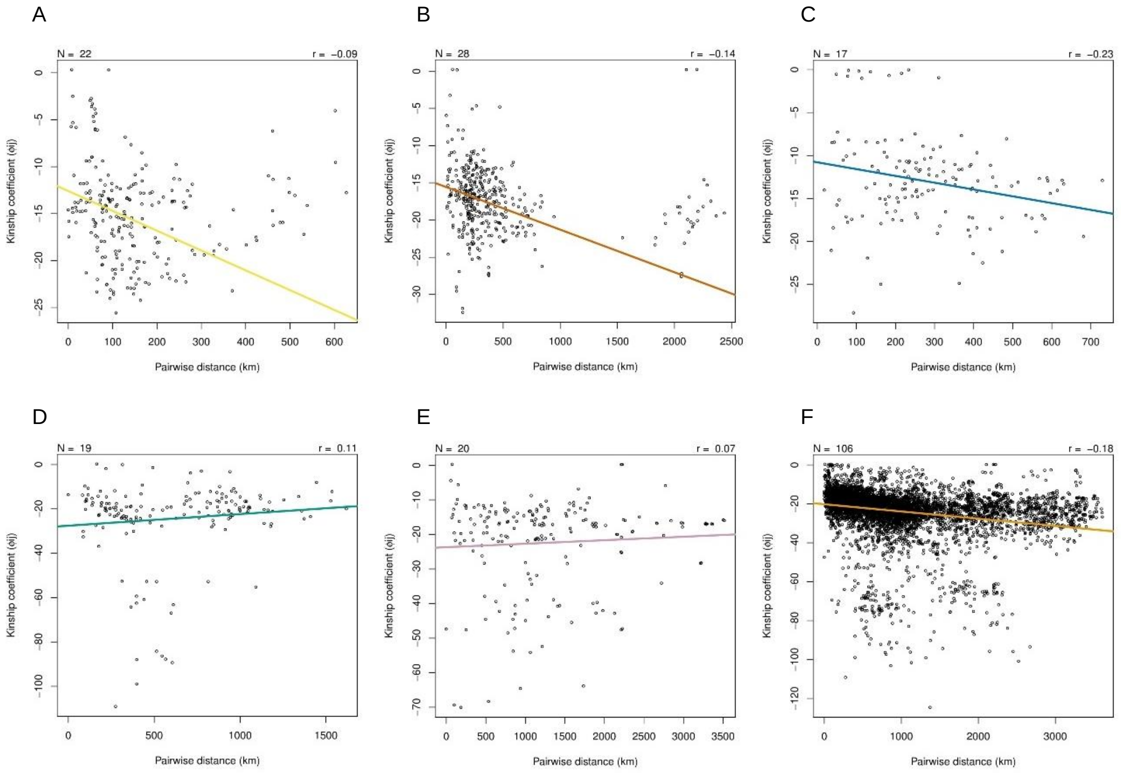

S6 Fig. Isolation by distance in the five genetic clusters of *O. glaberrima*. N = number of accessions in each population. r = Pearson correlation coefficient between the pairwise distances and kinship coefficients. Each dot represents the distance and relatedness between a unique pair of accessions within the population. Outliers are included. Samples falling outside the geographic of West Africa and samples without known coordinates are excluded. A, B and C represent the coastal populations. D and E represent the inland populations. F represents the entire West African population (N = 106) combined.
