# Supplementary figures and images for: Origins and geographic diversification of African rice (Oryza glaberrima)
Source: PLoS One. 2019 Mar 6;14(3):e0203508. doi: 10.1371/journal.pone.0203508 (PMC6402627; doi:10.1371/journal.pone.0203508)

*Phr1* 4: 23,491,785 – 23,494,110

*MOC1* 6: 17,930,149 – 17,931,657

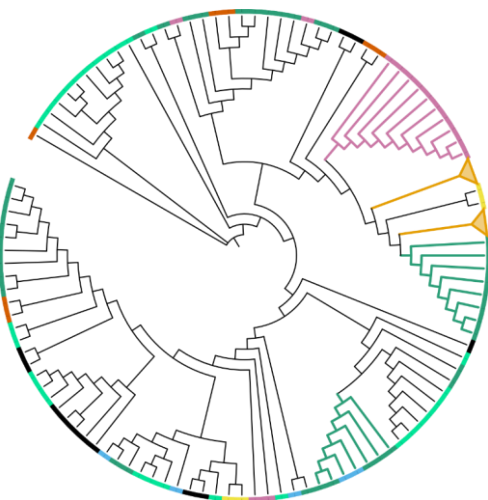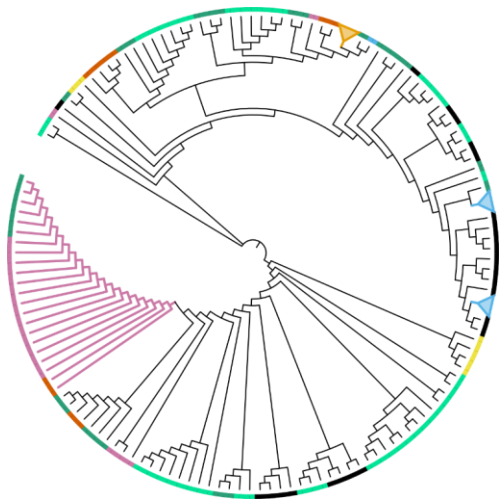

*Rc* 7: 5,268,358 – 5,270,384

*lpa1* 8: 19,078,252 – 19,082,033

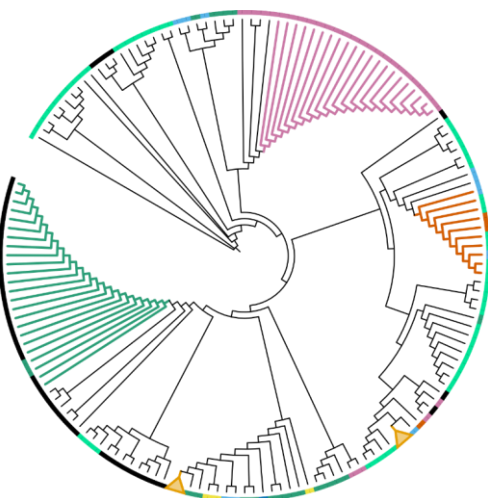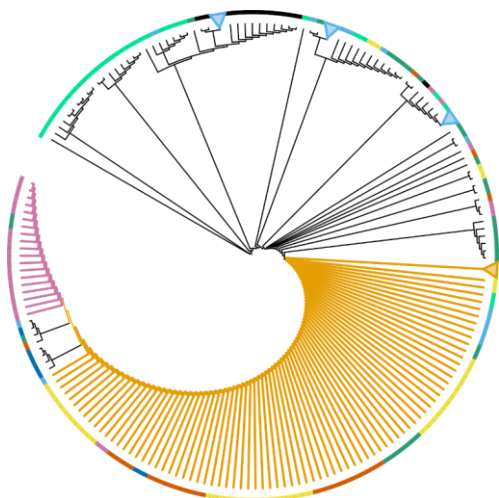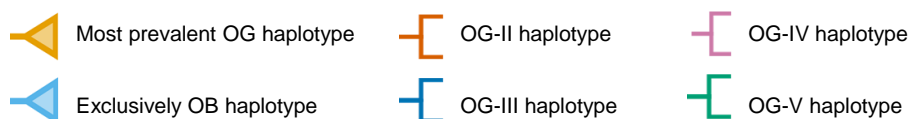

**S7 Fig. Separation of OG-IV haplotype in multiple domestication genes.**

Supplement: S7 Fig — (PDF) [file pone.0203508.s017.pdf]
