# Supplementary material for: Origins and geographic diversification of African rice (Oryza glaberrima)
Source: PLoS One. 2019 Mar 6;14(3):e0203508. doi: 10.1371/journal.pone.0203508 (PMC6402627; doi:10.1371/journal.pone.0203508)
Supplement: S8 Fig — (PDF) [file pone.0203508.s018.pdf]

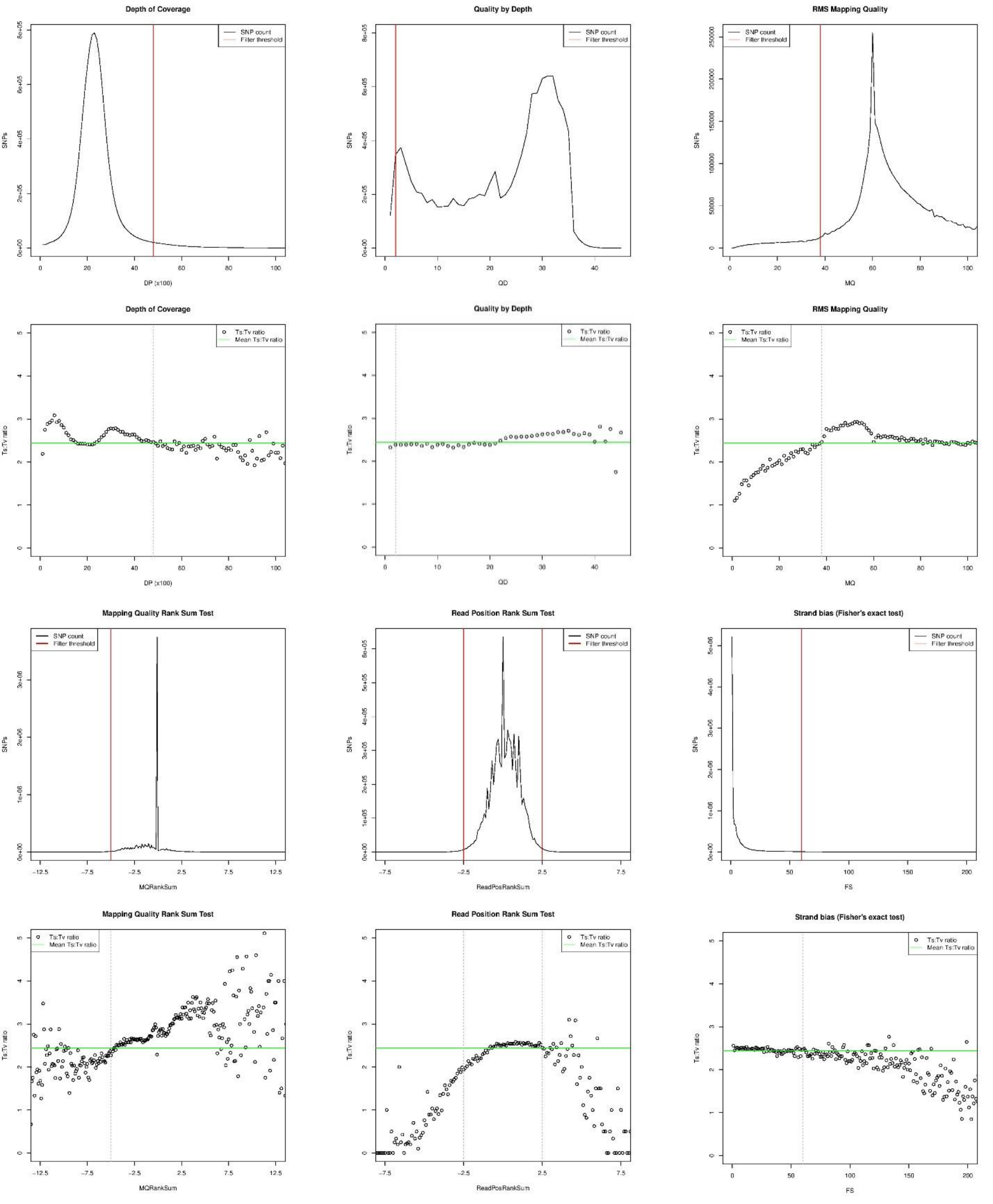

S8 Fig. SNP count and Ts:Tv ratio grouped by filter class. Filter thresholds are indicated in red and correspond to those applied in call set 1a. Mean Ts:Tv ratio before filtering was applied is indicated in green.
