# Supplementary material for: Origins and geographic diversification of African rice (Oryza glaberrima)
Source: PLoS One. 2019 Mar 6;14(3):e0203508. doi: 10.1371/journal.pone.0203508 (PMC6402627; doi:10.1371/journal.pone.0203508)
Supplement: S9 Fig — (PDF) [file pone.0203508.s019.pdf]

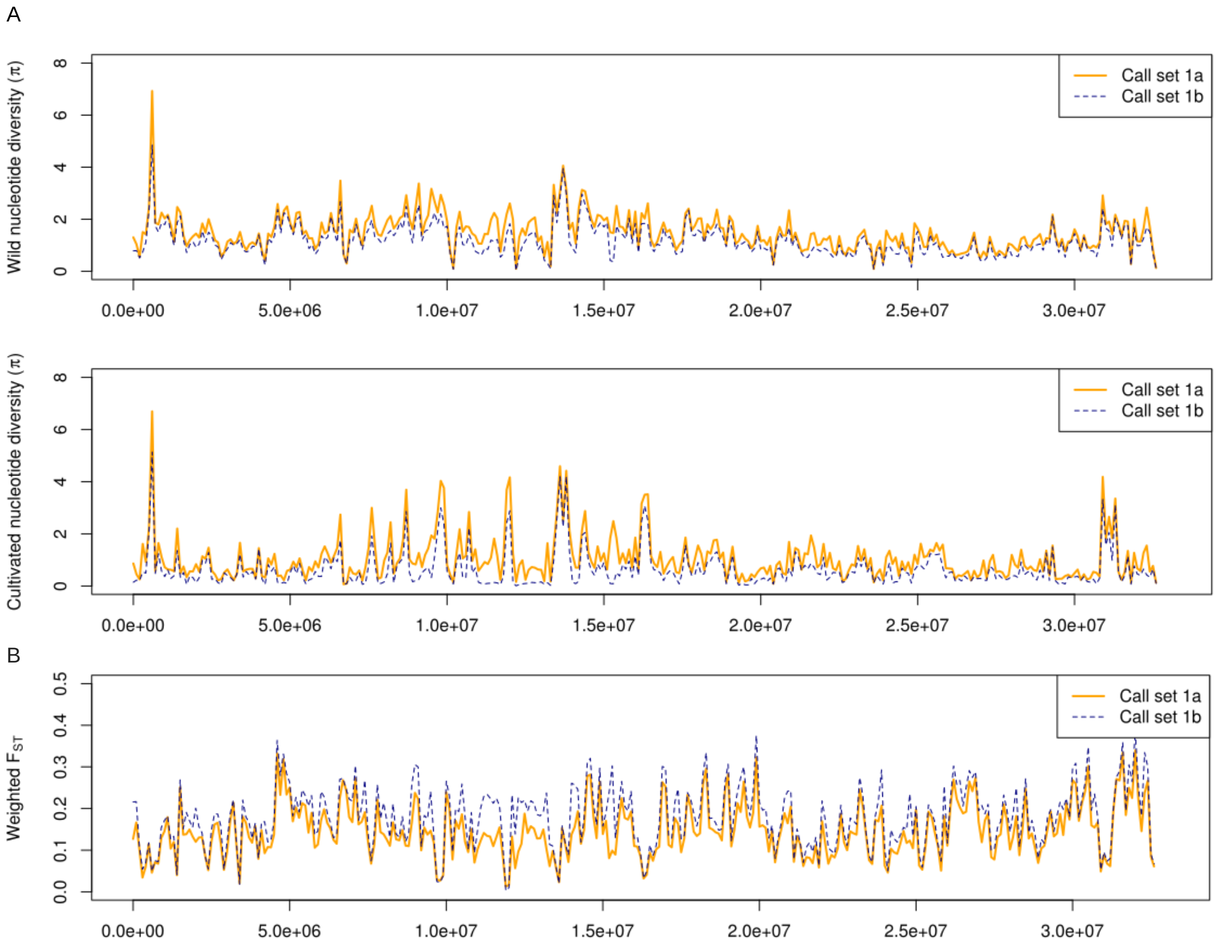

S9 Fig. Effect of filtering thresholds on nucleotide diversity and fixation index. For illustration purposes, only chromosome 1 is shown. A. Nucleotide diversity in both call sets. B. Fixation index in both call sets. As expected, nucleotide diversity is reduced in the stringent call set. F_{ST} is not markedly different. Considering that both statistics follow the same trend in both call sets, it was concluded that the effect of filtering on estimations of genetic diversity was merely one concerning absolute magnitude, and not concerning relative diversity.
